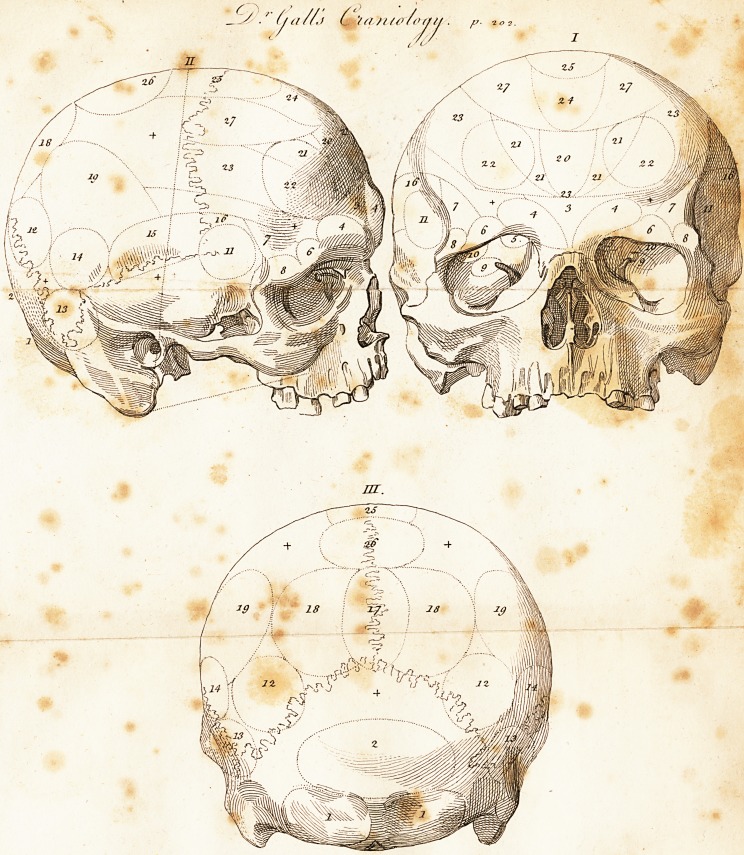# An Account of Dr. Gall's System of Craniology
*The Editors have been favoured with this interesting communication from a Correspondent at Dresden, and have judged it worthy of a place in the Medical Journal. For the further information of the public they think it, however, proper to state, that a full account of Dr. Gall's System has appeared in Germany, from the pens of Drs. Bishoff and Hufeland, and that a Translation of that work has been undertaken, and will shortly appear in London.


**Published:** 1806-03

**Authors:** 


Medical and Physical Journal.

VOL. XV.] MARCH, 1806. [NO. 85.

*Printed for R. PHILLIPS, by W. Thorne, Red Lion Court, Fleet Street, London.*

### AN ACCOUNT OF DR. GALL'S SYSTEM OF CRANIOLOGY.*

THE Craniology of Dr. Gall was the favourite topic of the German literati, during the summer of 1805, at almost every university and capital of the northern provinces of Germany. Dr. Gall, who is a native of Suabia, began his literary pursuits at the university of Strasburg, where he took the degree of a Doctor in medicine. From thence he visited Vienna, where he soon became a favourite of the public as an eminent physician. Every moment he could spare from his professional avocation, he employed in natural philosophy, and particularly in the researches of the conformation and anatomy of the human brain. His discoveries could not fail to attract public notice and observation; and he opened public lectures on his craniology, having previously obtained the sculls of many hundred animals and of men; in the protuberances and depressions of whom, his new hypothesis was always found to be true. The government, however, by the suggestions of some fanatical priests, took the alarm, and he was forbidden to continue his lectures. But this did not stop his inquiries; students in physic and men of research, came from every part to procure information, which he never refused, and his doctrine was soon spread all over Germany by the writings of some of his pupils. The Doctor himself prepared a work, illustrated with copper-plates, in which all his striking observations on comparative anato-

* The Editors have been favoured with this interesting communication from a Correspondent at Dresden, and have judged it worthy of a place in the Medical Journal. For the further information of the public they think it, however, proper to state, that a full account of Dr. Gall's System has appeared in Germany, from the pens of Drs. Bishoff and Hufeland, and that a Translation of that work has been undertaken, and will shortly appear in London.

my and the dissection of the brain were to be laid before the public. Subscriptions for it were opened, and completed in a short time. But previous to its publication, the Doctor resolved to make a circuit of all the northern universities and capitals of Germany, in order that the literati and professors might hear and scrutinize his Lectures, which he intended to deliver in every place wherein he should make any residence.

In the beginning of last spring the Doctor set out for Berlin, and lodged in the house of his intimate friend, Mr. Kotzebue; he here met with universal applause; the king, the queen, princes and princesses interested themselves so much in his discoveries, that he obtained an invitation to go thro' a course of lectures in the presence of the royal family, during which the queen inspected the dissection of a human brain, while the Doctor demonstrated the whole series of his astonishing discoveries. A rancorous attack was now commenced on his theory by Doctor Walter, first anatomist in Berlin, but it failed of the intended effect, every person being convinced that it was dictated by envy. On the opposite side, the justly renowned Dr. Hufeland, first physician to the king, almost all the faculty, as well as other proficients in that line of science, candidly professed their full assent, and several interesting tracts were published, in which ample justice was done to the theory.

Dr. Gall visited the houses of correction and prisons in Berlin and Spandau, and gave the most convincing proofs of his ability to discover, at first sight, such malefactors, thieves, and men of particular talents, as were amongst the convicts and prisoners; and a full and satisfactory account of those visits was published at Berlin, in a paper called the Plain Dealer. From Berlin he went to Dresden, where his lectures met with general approbation. On leaving that capital, he proceeded to Torgau, on the borders of the Elbe, whither the culprits of all Saxony are taken, in order to be put to work in a house of correction which is supported at the expence of the whole Electorate. In this journey, the Doctor was accompanied by several gentlemen of learning, particularly by Prof. Boettiger, now settled at Dresden, where he incontestibly proved by observations, that his Craniology was founded on the firmest basis. Mr. Boettiger has published these observations, an abstract of which is given in the following account; to which is subjoined a delineation of the organs, as they have been discovered by Dr. Gall's inquiries.

*A faithful*

*A faithful Account of Dr. Gall's visiting the House of Correction, and the Hospital of Torgau in Saxony, the 4th and 5th of July,* 1805.

Dr. Gall, soon after his arrival at Dresden, expressed a strong desire of visiting the principal houses of correction, hospitals, and charitable institutions of the Electorate of Saxony. Proper orders were therefore given by the governors to the stewards of the Bridewells of Waldheim, Torgau, and Zwickau to that purpose; but Dr. G. being prevented by other engagements from undertaking a journey to every one of these three places, contented himself with going to Torgau, to examine the two institutions established there, and to corroborate, by living examples, his psychological and craniological observations, in presence of several attentive and judicious witnesses.

The last mentioned town contains two establishments; in one of them about four hundred prisoners are kept for punishment ; in the other, almost as many lunatics and ideots for mere confinement; and both of them must certainly furnish the curious with various and copious matter for reflection. The sight of so many fellow-creatures, who are labouring under numberless physical and moral evils, must deeply wound any heart not divested of humanity; the idea, however, that every thing is done for their relief, affords a small degree of comfort.

Dr. Gall arrived, and several persons of distinction, friends as well as opponents of his doctrine, met, to be present at this curious and interesting investigation.

Mr. Wagner, one of the governors, had ordered the steward Seyfert, and the chaplain Reynel, both men of judgment and penetration, to make out a list of the most notorious felons and lunatics, describing at the same time, the reasons of their confinement, their characters, the state of their health, &c. according to which the examination of their sculls was to take place, and to which Dr. Gall's observations were instantly compared. He has neither seen this list before nor afterwards. Some particularly distinguished male felons were brought before him, one after the other, which he surveyed with the greatest attention, and delivered carefully his judgment upon them.

In P. a lock-smith from Gœrlitz, who was confined as a false coiner, and who was known in the house as being of a mechanical turn, he immediately discovered the decided sense for mechanics, which this man, according to his own account, had evinced even from his childhood. It

 would would have been natural to think, that according to the direction which this instinct took afterwards, and of which he made so bad a use, the sense for numbers might have been found likewise in him; but Dr. Gall did not see any thing of it; and at some trials, he proved to be but a poor accomptant. This circumstance confirmed his former observations on the organisation for mechanics, according to which, the mechanical skill may exist without the least notion of arithmetic and geometry.

In the prisoner A——, he observed less the instinct for thieving, than that of lust, with a violence, as an effect of his organ, for fighting. By comparing the list with his assertions, it was found, that he had been guilty of highway robberies, and a rape, and that he was subject to epileptic fits.

In S——, he observed besides the distinguished sense for thieving and that of carelessness, a remarkable skill for finding and remembering places. After enquiries, it was found, that he had often been employed as a messenger, and that he had discovered every place with the greatest ease.

Dr. G. was struck at the sight of T——, (a notorious fellow, who had formerly belonged to a gang of robbers) on account of his particular instinct for fighting. "This man," said he, "might have been a good soldier and freebooter." He discerned likewise in him the instinct for music, and it appeared that he had learned to play on the violin without any instruction.

Every person was desirous to know what Dr. G. would say about T——, who was known in the house as a thief full of cunning, and who having made several times his escape, wore an additional iron. It was surprising, that he saw in him far less the organ of cunning, than in many of the other prisoners. However it was proved, that examples and conversation with other thieves in the house, had suggested to him the plan for his escape, and that the stupidity, which he possesses, was the cause of his being retaken.

The Doctor examined with the utmost attention the prisoners which were confined for being incendiaries, as this crime is not to be met with in Austria, which is so very frequent in Saxony. He observed, that the inclination for it, which is said to be often almost irresistable, was the effect of several co-operating organs. In one of these incendiaries, who was about forty-nine years of age, he discovered the organs for cunning, for thieving, for finding, and remembering places easily, for recollecting words and and the flat and unfortunate organisation, which he says is adapted for every thing that is bad.

In another he met with the organ for thieving and cunning in a superior degree, and with all the other symptoms which characterize avaricious and envious people; but in none of them did he distinguish the instinct for murder, or any other organ leading to violence.

Whilst the observations upon single individuals were continued, the steward had ranged all the rest of the prisoners of both sexes into two separate ranks, for which purpose the large yard adjoining to the house was very convenient. The Doctor, with penetrating looks, walked through this very numerous company of thieves and rogues, making proper remarks upon every thing which he found particularly striking. In the whole number, at least seven out of eight had been brought there, for having committed greater or smaller thefts; and that no innocent person was amongst them, could be easily proved by their organs for thieving, which was seen or felt at the first look or touch. Even the most obstinate sceptics were obliged to yield to the convincing proofs of this organ. Whoever cannot read these characters, will hardly be able to read any thing which the finger of nature has written. But here Dr. Gall protested, in the most solemn manner, against all misinterpretations, by repeating several times, the restriction to those who were present, that though the organ for thieving was eminent in most of the prisoners, it would be wrong and dangerous to judge, that every person provided with the same organ must be a thief. It was however remarkable, that in most of the whole company of rogues, the organ for reflection, which is placed on both sides of the upper part of the scull, was scarcely to be seen at all; as, on the contrary, the organ of lust was eminently disclosed in many of them. The want of the former, makes careless beggars and vagabonds; and the stronger existence of the latter, leads often to pilfering and cheating, particularly in servants.

Whenever Dr. G. met with some organisations either very fortunate or unfortunate, he did not fail to make judicious observations upon them, and ordered those, whose sculls and organs were similar, to stand next to each other, that they might be readier for inspection and comparison. This happened with four particularly distinguished heads, whose depressed and flat sculls seemed to shew something particularly sullen and beastly. Dr. G. took this opportunity to explain, how these people might have

 been been assisted by an early education, and by a prudent choice of their profession. However, he, and Dr. Spurzheim, his fellow traveller, declared, that besides these four very unfavourably formed heads, there was in the whole number not another to be found, who had such a dejected and distorted face and scull, as he had generally met with at the prisons and bridewells of Berlin, and that an exception here was a rule there. It was surprising to him, to find so many prisoners at Torgau, who were confined *ob delicta carnalia*, as in the Austrian and Prussian dominions, crimes of this sort are not so severely punished. Some very favourably organized persons justified their exterior at a minute's investigation. "Why did you resist so obstinately to your superiors?" exclaimed Gall, at the first sight of a man, who was only punished for seditious speeches, and obstinacy; and really the upper and back part of his scull, where Dr. G. finds the organ of the desire for glory and steadiness, which degenerates so often into obstinacy, were uncommonly prominent. In another he met with the organs of wit and arts, and called that construction a very happy and *home* rising one, which was to be found in great poets. The man was really notorious for wit; and a person who had known him before his confinement, assured us, that he recollected his making verses *extempore*. Besides the organ of thieving and cunning, which shewed itself, in spite of every disguise, no other distinguished itself so much as that of representation, which, according to Gall's doctrine, is the share of actors, mimics, and witty fellows.

Thus ended this interesting inspection of sculls and organs, which is highly instructive to the observation of man, and to the philosophical physician. We lost much by the absence of the aged and experienced physician, Dr. Michaelis, who could have explained many things, which appeared to us enigmatical. This loss was more severely felt, at our visiting next the female ideots and lunatics. What a field for a psychological physician and anatomist! In the lower rooms of the right wing there were few to be found, in proportion, whose state required to be put in chains. It had already been resolved to introduce more generally the use of strait waistcoats. Only two of them were in violent paroxysms, but they shewed by their language the cause of their misery, that is to say, an extravagant lust. One of them suffered Dr. Gall to touch her scull, and this produced the usual effect of her disordered instinct. It was observed, that the most frequent quent effect of frenzy was the volubility of the tongue, and an inclination for quarreling, which they shewed by breaking every thing in their reach, and by tearing to pieces their clothes, if they had got their hands at liberty. In two persons, who distinguished themselves particularly in that manner, the corner behind, and that below the *os bregmatis,* was found particularly prominent. In a female lunatic, who usually sits silent on the lower part of a window, and who fancies she looks down from Koenigstein* and to lie in the page's bed,† all those, who were made attentive to it, discovered the organ of finding and recollecting places conspicuously disclosed. As we returned across the yard, Dr. G. saw several ideots leaning against the wall, and basking there. Two sisters amongst them, of the name of Bolutchen, showed rough and unmannerly demonstrations of joy at the sight of some feathers, which an officer of cavalry, who was of our company, wore in his cap. Dr. G. took this opportunity, to make some very judicious observations concerning the confinement of these wretched creatures.

Towards the evening the Electoral hospital for the poor and orphans, situated in another part of the town, was inspected, where three hundred and forty-two poor persons and orphans are supported. Under the name of poor are understood ideots, melancholists, cripples, and persons affected with some incurable disease. The orphans are provided for after the fifteenth year of their age, the boys being apprenticed, and the girls put to service; only those children who are incapable of any other destination, are kept in the house amongst the poor. The hospital contained thirty incurable blind persons, fourteen others, born deaf and dumb, and many more, who, on account of their natural stupidity, made every attempt of instruction unsuccessful, but could nevertheless furnish materials to many physiological and craniological reflections. Dr. G. was struck at the sight of a man born blind, of the name of Grellman, a very assiduous instructor of the orphans, as he discovered in him a peculiar organ for mechanical

 arts,

* Koenigstein is a fortress situated upon a high rock, about twenty-four miles from Dresden, near the river Elbe, and the frontiers of Bohemia.

† The page's bed is a narrow projecting part of the steep rock, upon which the fortress of Koenigstein is built. It is related, that many years ago, a page of the King of Poland, in a drunken fit, went to sleep upon it. The King ordered him to be fastened with ropes, to prevent his falling down an immense precipice.

arts, and who spends his leisure hours in making bird-cages, and other artificial works, to which the measure of proportion is particularly required.—The existence of the very prominent organ for music in a person deaf and dumb was extremely surprising. No one had paid attention to it till then, and the question arose, how this organ could shew its effects in a deaf and dumb person? After inquiry, it was found, that he used to do every thing by time, and that he was not quite insensible to the sound of a drum.

In Gerish, a melancholist, the organs of theosophy, and that of representation, were strongly marked. He was known in the whole house as a visionist, and a fanatic. He related to us, that spirits often visited him, and told him stories of an extraordinay nature. Dr. G. found a great resemblance between him and a theosophical shoe-maker, whom he had seen in Dresden. In another blind man he met likewise with an elevation of the organ of theosophy, and, we were told, that he was fond of listening to the reading of theological works. In another, he observed that the organ of being good-natured was very visible at the upper part of the front of the scull, as he had found it in a servant of Baron Kalkhof at Vienna, whose scull was the first he had seen of the same construction, and who was represented to him as an extraordinary good-natured man. The former had been blind since the first fortnight of his birth, and yet the organ of finding and recollecting places manifested itself strongly in the corners of his eye-brows. To Dr. G s question, if he used to dream often? he answered, that he did it always; and being asked, of what he dreamed? he said, that he held mostly conversations of foreign countries in his dreams; and that at present the republic of the seven islands filled his mind more than any thing else. His principal delight is, to hear any person read the newspapers. In a young man half grown, Dr. G. discerned immediately, what he calls the organ of murder in a strange degree; and to his great surprise he was told, that both his parents had been found guilty of being incendiaries. He recommended a partieularly strict vigilance over him. One of the rooms contained four beings, which could hardly be called human, as their beastly stupidity, their grinning, and their staring eyes, shewed the greatest insensibility. One of them was particularly shocking, as his prominent eyes, and the whole form of his head, signified a *hydrocephalus*. He eats stones, and his own excrements, crements, if he can do it without being noticed. Dr. G. said, that unfortunate cseatures of this sort had often retired to forests, and supported themselves by following their beastly instincts, and that therefore, many fabulous accounts of wild men could be explained. There was, in general, no want of matter for reflections to be made upon watery-heads, or dropsy in the brain, as this distemper was to be met with there in all its degrees. In a man, whose head was of an unnatural form, Dr. G. was doubtful, if he should explain the remarkable elevation above his ear as an organ of cunning, or as an effect of his having a watery-head. He said, that such a head might contain two pounds of water! In a patient, who chatters continually, and speaks in violent terms of his being cheated by his relations, Dr. G. found the organs at the upper part of the front bone, which seem to be necessary for the imagination of a poet, very eminent, mentioning at the same time, that most of the fanatic talkers are distinguished by these organs.

We next visited the rooms where disordered and lunatic females were confined. In a quarrelsome woman, Dr. G. observed, that the organ of fighting and that of vanity were particularly disclosed. In another, he desired us to notice the amorous and voluptuous gestures of the head, of which he had spoken in his lectures, and the behaviour of this person completely confirmed his assertion.

The following morning several persons, whom Dr. Gall had reserved for a more minute investigation, were brought before him in the committee room, one after the other, that he might practise his organoscopy, being less interrupted by a croud of spectators. One of the company undertook to keep a formal protocol, which was read to the Doctor after the bussiness was finished, either for his confirmation or rectification. The contents of it are as follows:

Elizabeth Wedekind appears, who is confined on a suspicion of murder till she can prove her innocence. The Chaplain observes, in his list of the prisoners, that she possesses the cunning of covering herself with the cloak of piety and devotion. She repeated with great eloquence the protestations of her innocence. The organ of murder was found on her in a very small degree; as, on the contrary, the organ of talking shewed itself more, and still more that of cunning. Neither the organ of ambition, nor of vanity, nor of highness was visible. She has been pregnant twelve times, and delivered seven times. The construction construction of her scull is a favourable one, particularly on account of the organs placed on her forehead.

A woman, of the name of Weber, being in the greatest despair, as a reception was refused to her every where, threw her child, four years of age, into the water, and without being moved by its pitiful cries, she left it to be drowned. Dr. G. did not know any thing of her, but that she had murdered her child. He observed in her as follows:

At the hinder part of her head, which is very flat, the organ of love for her children is not disclosed; but the want of it is not so great as he has seen it in three female convicts, who were guilty of having murdered their children, and whom he had an opportunity of examining very strictly at Vienna, Spandau, and Leipsig. Her organization is very different upon the whole; only the organ of retaining words is very visible. That of murder is not to be discovered in her. The want of the necessaries of life, however, probably brought her to the dreadful resolution of committing murder, as appears by the written proceedings of her trial. She was the only female convict that we saw this and the preceding day who shed tears, and that shewed an undisguised repentance. Her declaration, which was confirmed by the chaplain, that she never had any education, that she had only learnt to read while in the bridewell, and that she had got many sentences of the bible, and several psalms, by heart, deserve to be noticed, to justify Dr. Gall's opinion of her.

Three complete thieves appeared. One of them, of the name of Weber, was examined first. " Two of his organs are disclosed, both in an extraordinary manner, that of thieving with an uncommon cunning, and that of representation." Of the former, the account of his life furnished us with convincing proofs, and of the latter he gave us instantly an example, by representing an enraged person in the most natural manner. He has played several parts, which have done credit to his abilities as an actor. At the same time it was told of him, that he could deliver any sermon he had heard, and imitate exactly the voice, gesticulation, and declamation of the preacher.

Dr. Gall said of M**r, a journeyman bricklayer, as follows: " His organ for thieving is very visible; he has likewise the organ of representation; but his organ for highness or obstinacy, and that for music, are still more eminent." Upon enquiring into his conduct, we were assured, that he was very obstinate and rebellious, and that he he had once made his escape. In respect to his instinct for music we were told, that he was the best psalm-singer in the whole congregation. Dr. G. observed, that the two latter convicts ought to be severely punished, as their organisation was so very favourable. In M. Dr. Gall found the decided organ of thieving, and a great carelessness; the organs of reflection not being formed at all." We were much surprised to be told, that this man, in whom Dr. G. had not discovered the organ of representation, possessed extraordinary abilities in imitating the voice of animals; but we were convinced, after enquiries, that his talent was not a natural one, but acquired by study. He related to us, that when he was a Prussian soldier, garrisoned at Berlin, he used to deceive the waiting-women in the foundling-hospital, by imitating the voice of exposed infants, and counterfeited sometimes the cry of a wild drake, when the officers were shooting wild ducks. His art seems to proceed from a sort of ventriloquy, as, during his practice, the muscles of his lips are scarcely moved.

Two other thieves were examined, and in both of them the organs of thieving and that of cunning were remarkable. Of one of them, he expressed himself thus: "To judge by the flatness of the fore part of the scull of this man, he is venal, and easily to be seduced, and his organ for music is eminent." The man spoke much of his being thrown into his misfortunes by seduction. In respect to music, he acknowledged that he joined with pleasure in psalm-singing. Two others were brought before him. Of one of them, he said: "The organ of good-nature is not visible in him; but his organ of lust is strong, and contributes to the disclosure of the organ of thieving." The man's confession, that he was very fond of the fair sex, confirmed partly Dr. G's observation. Of the other, he said: "His head is a pattern of inconstancy and confinement, and there appears not the least mark of the organ of courage." This cunning rogue has been able to gain a great authority amongst his fellow convicts. How is this to be reconciled with the want of constancy, which his organisation plainly indicates? Dr. G. answered: he had got his authority by cunning, not by courage. We were told, that when he was apprehended, he lost all countenance, and neither knew what to say, nor what to do.

Amongst many others, who were produced, the organ of representation was particularly disclosed in a shoemaker. The account he gave of himself, with his gesticulations, attracted the attention of the whole company. He imitated the the mien of his neighbour in a striking manner; he represented an enraged person surprisingly well; and told us, that at the usual meetings of shoemakers, he had always been the merry-maker. A watchmaker gave an opportunity to Dr. G. to make some observations on the organ of poetry, which he discovered in him, and which he considers as the continuation of that of music, in an oblique direction in the corners of the forehead.

A man of the name of Kœlner was confined in Bridewell, on account of some threatening speeches to a person, who was afterwards found murdered. Dr. G. discovered in him the organ of obstinate highness, and that of courage, but by no means that of murder. However, according to his opinion, the organs which he had noticed in him, were sufficient to lead any one to commit murder. He observed in another person, that the fore-part and hind-part of the scull were highly arched. Dr. G. had formerly the opportunity of meeting with sculls of the same construction in Generals, who had been advanced from private soldiers to their high rank by merit and heroic actions. A taylor, from Dresden, a cunning thief, who had once effected his escape from bridewell, was the last who was introduced to Dr. G. for examination. He said of him, as follows: "The upper part of the front bone is flat, and consequently the organ of good-nature is disclosed but very little. The organ of cunning is eminent; but the organ of highness very seldom met with in thieves, is particularly striking." As we entered afterwards into conversation with him, he gave us to understand, with a great deal of presumption, that he did not intend by any means to follow his trade as a taylor, as he was able to perform by moon-light, what others did by day-light. The account of the thefts he committed proves, that he was prompted to them by vanity, as he always wished to appear greater and higher than he was, and of course the organ of highness was very active in him. Dr. G. before his departure, visited the male ideots and melancholists, in the left wing of the building. He met there with various matter for judicious observation. He proved, for instance, concerning watery-headed ideots, that in spite of the inactivity of almost all their organs, they shewed a great lust, because the instinct for the other sex in the small brains suffers the least by the water in the head. Some proud fools distinguished themselves, particularly one, for instance, who fancies himself Elector of Saxony, and another who pretends that the cities of Amsterdam and Hamburgh are to honour his drafts. In both of them the elevation elevation of the skull in the middle of the *sutura sagittalis* is very remarkable. Dr. G. gave likewise some hints how to treat fanatics, by using topical remedies and poultices if the distemper should not be inveterate.

*Organs of Dr. Gall's System of Craniology.*

1. The organ of lust, or of mutual instinct of both sexes. Fig. II. III.
2. The organ of a person's love for his children, or of an animal for his young ones. Fig. II. III.
3. The organ of being tractable in education, *memoria realis*. Fig. I. II.
4. The organ of finding, and remembering places. Fig. I. II.
5. The organ of recollecting persons (in the eye-hole.) Fig. I. II.
6. The organ of recollecting and comparing colours. Fig. I. II.
7. The organ of music. Fig. I. II.
8. The organ of arithmetic. Fig. I. II.
9. The organ of finding, and remembering words (in the eye-hole). Fig. I
10. The organ of language (in the eye-hole.) Fig. I.
11. The organ of mechanical arts. Fig. I. II.
12. The organ of friendship and attachment. Fig. II. III.
13. The organ of fighting. Fig. II. III.
14. The organ of murder. Fig. II. III.
15. The organ of cunning. Fig. II. III.
16. The organ of thieving. Fig. I. II.
17. The organ of highness. Fig. III.
18. The organ of thirst for glory, and that of vanity. Fig. III.
19. The organ of reflection. Fig. II. III.
20. The organ of comparing judgment. Fig. I. II.
21. The organ of philosophical judgment (includes No. 20.) Fig. I. II.
22. The organs of wit. Fig. I. II.
23. The organ of the power of induction (includes the organs No. 20, 21, and 22.) Fig. I. II.
24. The organ of being good-natured. Fig. I. II.
25. The organ of theosophy. Fig. I. II. III.
26. The organ of constancy. Fig. II. III.
27. The organ of representation (includes No. 24.) Fig. I. II.

** Are places not marked.

## Figures and Tables

**II I III. f1:**